# Latent tuberculosis infection and infection-associated risk factors for miner workers with silicosis in eastern China

**DOI:** 10.1186/s12890-024-02985-z

**Published:** 2024-04-15

**Authors:** Xinsong Hu, Cheng Chen, Qianqian Gao, Lang Zhou, Yan Shao, Guoli Li, Honghuan Song, Qiao Liu, Lei Han, Limei Zhu

**Affiliations:** 1 School of Public Health, Nanjing Medical University, Nanjing, China; 2 Department of Chronic Communicable Disease, Center for Disease Control and Prevention of Jiangsu Province, Nanjing, China; 3 Department of Occupational Disease Prevention, Center for Disease Control and Prevention of Jiangsu Province, Nanjing, China

**Keywords:** Tuberculosis, Latent tuberculosis infection, Silicosis, Risk factors

## Abstract

**Objectives:**

Silicosis people are at high risk of developing pulmonary tuberculosis. Whether silica exposure increases the likelihood of latent tuberculosis infection (LTBI) was not well understood, and potential factors involved in LTBI risk among silicosis people were not evaluated before. Thus, LTBI among silicosis people and potential risk factors for LTBI among silicosis people were evaluated in this study.

**Methods:**

A cross-sectional study was undertaken for 130 miner workers with silicosis. The QFT-GIT was performed for LTBI detection.

**Results:**

The LTBI was high to 31.6% (36/114) for silicosis participants, and 13.1% (13/99) had a history of tuberculosis. Drinking was associated with LTBI risk (OR = 6.92, 95%CI, 1.47–32.66, *P* = 0.015). Meanwhile, tunneling work was associated with an increased risk of LTBI compared with other mining occupations (OR = 3.91,95%CI,1.20–12.70, *P* = 0.024).

**Conclusions:**

The LTBI rate of silicosis participants was high and more than 10% had a history of tuberculosis. Drinking alcohol and tunneling were independent risk factors for LTBI in silicosis participants.

## Introduction

Silicosis is a fibrotic lung disease caused by inhalation of free crystalline silicon dioxide or silica [1]. While silicosis is the most main and severe occupational disease in China. From 1990 to 2019, new incident silicosis cases in China accounted for more than 67% of the world’s silicosis cases each year, and the prevalent silicosis cases were greater than 80% of the silicosis cases in the world [2]. When people are exposed to coal dust or silica dust for a long time, their respiratory system is severely damaged, resulting in pneumonia, pulmonary interstitial fibrosis, pulmonary function decline, and immunity weakened. As a result, people will be easily infected by *Mycobacterium tuberculosis* (MTB) and other various viruses. Tuberculosis is not only the main comorbidity of silicosis, but also the main cause of death for silicosis patients [3, 4]. China is one of the high-burden countries of tuberculosis in the world, and the number of tuberculosis in China ranks third among the 30 high-burden countries after India and Indonesia [5]. In 2021, the number of new TB patients in China was nearly 780,000 [5]. Meanwhile, latent tuberculosis infection (LTBI) is a persistent immune response to MTB infection without clinical symptoms or imaging features. It was estimated that a quarter of the world’s population was infected with MTB, and 5-10% of them will develop active tuberculosis [6]. How is the prevalence of LTBI among silicosis was rarely reported, one study indicated that the rate of LTBI in pneumoconiosis patients was as high as 66.4% with a small sample size [7]. In 2015, WHO proposed a global strategic goal to end the tuberculosis epidemic (END TB) by 2035 and recommended LTBI testing and preventive treatment for silicosis people in high-burden countries [8, 9]. In this study, we aimed to investigate the current situation of LTBI among silicosis people from mining, and explored potential risk factors for LTBI among this vulnerable population.

## Methods

### Study design and population

A total of 130 retired coal workers with confirmed silicosis attending the annual health examination were enrolled in this study in Datun Town, Xuzhou City, Jiangsu Province in April 2023. In this study, silicosis was classified into four stages: early stage, stage 1, stage 2, and stage 3. The classification of silicosis was defined according to the International Labour Organization (ILO) [10]. Participants with uncertain LTBI results and invalid questionnaires were excluded. The study was approved by the institutional review board of Jiangsu Provincial Center for Disease Control and Prevention, informed consent was obtained from each participant, and we confirmed that all experiments were performed in accordance with relevant guidelines and regulations.

### Measures and definitions

For each participant, a questionnaire survey was conducted by a trained person through a face-to-face interview. The questionnaire consists of two parts: the first part includes general demographic characteristics, such as smoking, drinking, tuberculosis contacting history, etc.; The second part is the occupational factors, such as silicosis grading, work categories, ventilation at work, etc. Digital chest radiography was performed on all participants to exclude active pulmonary tuberculosis. Blood of each participant was drawn for QFT-GIT testing. Samples with indeterminate results in the first experiment would be tested again, and samples with indeterminate results in the second test were excluded from the analysis. Body mass index (BMI) was classified as lower weight (< 18.5 kg/m^2^), normal weight (≥ 18.5 kg/m^2^ and < 24.0 kg/m^2^), overweight (≥ 24.0 kg/m^2^ and < 28.0 kg/m^2^), and obese (≥ 28.0 kg/m^2^) [11]. Smoking status was classified as never smoking, smoking cessation and current smoking. Those who quitted smoking for at least six months were defined as not smoking, and those who abstained drinking for at least six months were defined as not drinking. Work categories were divided into mining and tunneling, and other types. The miners were defined as people who worked underground to collect the coal, and tunnellers were people who dug and repaired the tunnels.

LTBI was determined by the positive results of QFT-GIT test. 5% of the samples were randomly selected to repeat for consistency, and all results were 100% consistent with the primary results.

### Data analysis

We compared between-group demographics using Pearson χ^2^ test or fisher exact test for categorical data. Multi-factor logistic regression was used to analyze the independent risk factors of LTBI in silicosis participants. Associations were described as adjusted odds ratio (OR) and 95% confidence interval (CI). The Kruskal-Wallis test was used to compare the quantitative QFT results of different groups if it was applicable. Statistical analysis was performed using SPSS 20 (IBM, Chicago, USA).

## Results

A total of 130 workers with silicosis disease were enrolled. Sixteen persons with indeterminate QFT-GIT results were excluded, thus 114 participants were finally included for LTBI analysis. We found 31.6% (36/114) persons had a positive QFT-GIT result. Because 15 persons only took QFT-GIT test but they did not join the questionnaires, so 99 participants with silicosis provided their demographic information, and the characteristics of the participants were shown in Table 1.

**Table 1:** Baseline characteristics of study participants

Characteristics		Total number *N* = 114	QFT Positive *N* = 36	%	QFT Negative *N* = 78	%	*P*
Age							**0.702**
	< 76	54	18	33.3	36	66.7	
	> 76	60	18	30.0	42	70.0	
BMI							0.152
	Normal	36	12	33.3	24	66.7	
	Overweight	44	18	40.9	26	59.1	
	Obese	19	3	15.8	16	84.2	
Education							0.916
	Primary school and below	49	16	32.7	33	67.3	
	Junior high school	38	12	31.6	26	68.4	
	Senior high school	10	4	40	6	60	
	College or above	2	1	50	1	50	
Residence							0.308
	Urban	85	30	35.3	55	64.7	
	Rural	14	3	21.4	11	78.6	
Smoking status							0.743
	Never	27	9	33.3	18	66.7	
	Ex-smoker	40	12	30.0	28	70.0	
	Smoker	31	12	38.7	19	61.3	
Drinking status							**0.047**
	Never	22	4	18.2	18	81.8	
	Abstainer	39	11	28.2	28	71.8	
	Drinker	38	18	47.4	20	52.6	
Tuberculosis contacting history							0.460
	No	93	30	32.3	63	67.7	
	Yes	4	2	50	2	50	
Tuberculosis disease history							0.833
	No	86	29	33.7	57	66.3	
	Yes	13	4	30.8	9	69.2	
Diabetes							0.879
	No	67	22	32.8	45	67.2	
	Yes	32	11	34.4	21	65.6	

All 114 participants were male, and 85.1% (97/114) participants were older than 65 years old. 36.4% (36/99) people had a normal weight, 44.4% (44/99) people were overweight, and 19.2% (19/99) of them were obese. 49.5% (49/99) of the participants had primary school education or below, while 31.6% (31/98) and 38.4% (38/99) of the participants were smoking and drinking, respectively. 6.1% (6/99) had close contact with tuberculosis, 13.1% (13/99) had a disease history of tuberculosis; 32.3% (32/99) had diabetes.

The results demonstrated that the LTBI rate was similar among those aged above 76 years old and below (33.3.0% vs. 30.0%, *P* = 702). The LTBI rate was higher among current smokers (38.7%) than in the other two groups who never smoked and those who stopped smoking (33.3%, and 30.0%), though the difference reached no statistical significance(*P* = 0.743). However, LTBI rates from never drinking, abstaining from drinking to currently drinking were gradually increasing, and the difference reached statistically significance (18.2%, 28.2%, and 47.4%, *P* = 0.016).

The characteristics of the occupational factors for the participants were shown in Table 2. In the classification of silicosis, 77.5% (86/111) of the participants were stage one silicosis. The work categories were majorly divided into coal mining (31.9%) and tunneling (59.3%). 71.2% (79/111) of the participants had been working for more than 25 years, and 80.2% (81/101) of them carried protective air equipment at work. We found that the LTBI rates among people with stage one (33.7%), two and three (38.5%), were all higher than the early-stage group (16.7%), respectively. In different work categories, the LTBI rates among tunneling workers (41.8%), mining workers (19.4%), and others (10.0%) showed a statistically significant difference (*P* = 0.020).

**Table 2:** Silicosis occupational factors of the study population

Characteristic		Total number *N* = 114	Positive *N* = 36	%	Negative *N* = 78	%	*P*
Silicosis categories							0.440
	Early-stage	12	2	16.7	10	83.3	
	Stage 1	86	29	33.7	57	66.3	
	Stage 2 and 3	13	5	38.5	8	61.5	
Work categories							**0.020**
	Others	10	1	10.0	9	90.0	
	Mining	36	7	19.4	29	80.6	
	Tunneling	67	28	41.8	39	58.2	
Work Ventilation condition							0.959
	No	19	6	31.6	13	68.4	
	Yes	87	28	32.2	59	67.8	
Occupational health education							0.518
	No	48	14	29.2	34	70.8	
	Yes	57	20	35.1	37	64.9	
Wearing Protective Equipment							0.473
	No	20	5	25.0	15	75.0	
	Yes	81	27	33.3	54	66.7	
Year of Silicosis exposure (years)							0.866
	< 25	32	10	31.2	22	68.8	
	>=25	79	26	32.9	53	67.1	

We performed a multivariate analysis for potential risk factors for LTBI among people diagnosed with silicosis. As shown in Table 3, current drinking was associated with LTBI compared to never drinking (OR = 6.92, 95%CI, 1.47–32.66, *P* = 0.015), and tunneling work was associated with an increased risk of LTBI compared to the mining jobs (OR = 3.91,95%CI,1.20–12.70, *P* = 0.024). Age, smoking, tuberculosis disease history and silicosis grade were not found in association with an increased risk of LTBI.

**Table 3:** Multivariate logistic regression analyses of risk factors for LTBI in silicosis people

Characteristic		OR(95%CI)	*P*
Age	< 76	Reference	
	≥ 76	0.88(0.35–2.24)	0.790
Smoking status	Never	Reference	
	Ex-smoker	0.40(0.10–1.54)	0.181
	Smoker	0.71(0.18–2.70)	0.613
Drinking status	Never	Reference	
	Abstainer	3.16(0.65–15.30)	0.153
	Drinker	6.92(1.47–32.66)	0.015
Tuberculosis disease history	No	Reference	
	Yes	1.48(0.26–8.48)	0.657
Silicosis categories	Early-stage	Reference	
	Stage 1	2.82(0.45–17.82)	0.270
	Stage 2 and 3	2.34(0.20-27.83)	0.502
Work categories	Mining	Reference	
	Tunneling	3.91(1.20–12.70)	0.024
	Others	0.78(0.07–8.95)	0.839

**Fig. 1:**
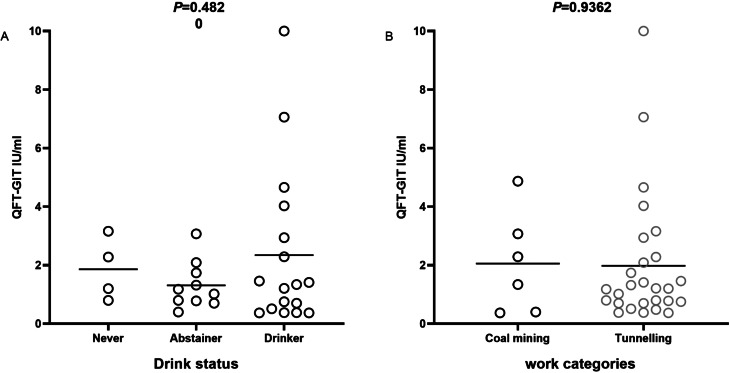
Distribution of quantitative QuantiFERON-TB Gold In-Tube (QFT-GIT) results among LTBI. (A) Distribution in different drinking groups (B) Distribution in different work categories

We further evaluated the magnitude of QFT TB antigen levels for LTBI among personal characteristics as well as silicosis occupational factors. Because the distribution of QFT TB antigen values was not in nominal distribution, the median value was adopted to show the average level of QFT TB antigen. We found the average QFT-GIT TB antigen levels of people of currently drinking and coal mining was higher, but there was no statistical difference (*P* = 0.4820, *P* = 0.9362, Fig. 1).

## Discussion

In this cross-sectional study for LTBI among silicosis participants, it was found a high LTBI rate among this specific population. LTBI rates among silicosis people with stage one, two and three were higher than the early-stage group. Meanwhile, in different work categories, the LTBI rates among tunneling workers was higher than the mining workers and others. Also, we found drinking and tunneling work were independently associated with increased risk of LTBI for silicosis participants.

There was a controversial on which laboratory method would be better for LTBI detection for silicosis people. In 2009, a study conducted in Hong Kong proposed that T-SPOT was more applicable than TST in silicosis people [12]. Meanwhile, studies had shown that T-SPOT had the advantages for LTBI detection with high sensitivity and high negative predictive value among silicosis participants [13, 14]. Meanwhile, a previous study had revealed similar effects of QFT and T-SPOT for the elderly underground coal miners [15]. Thus, IGRA-based method seemed to be a better method in the detection of LTBI among silicosis people, thus QFT was employed for LTBI detection in our study.

According to the study by Gao et al. conducted in 2015, the LTBI rate among the elderly aged above 60 years old in rural China was about 17.01% [16], while the LTBI rate of those silicosis from rural areas in our study was about 21.4%, which was higher than the corresponding age group people in rural China. This proved that silicosis people were at a high risk of latent tuberculosis infection. Several studies demonstrate LTBI for silicosis people in different settings. The infection rate of silicosis population conducted in Zhejiang, China was about 50.6% [17], while another study conducted in German showed that the LTBI rate for silicosis people was around 46.6% [15]. A study in Iran showed the LTBI rate was as high to 70.2% for the silicosis patients [18].

On one hand, we conducted a survey based on the physical examination of retirees, which suggested that these silicosis participants were relatively healthy, and without severe health problems [19]. More importantly, grade one silicosis accounted for the majority of the participants, which means people with more severe lung injury and silicosis with multiple complications would not participate in this physical examination.

In this study, we found one eighth of the silicosis participants had a history of tuberculosis disease. It was well-known that silicosis exposure would increase the risk of tuberculosis, and previous study had approved increased risk of tuberculosis for silicosis exposure [20]. Meanwhile, how many of them will develop tuberculosis was various across different regions, which depending on various factors, such as the exposure level of silicosis [21], TB prevalence [22], and individual health conditions.

There was an interesting finding in our study, the LTBI rate was low to 1/3 of those people with previous history of tuberculosis, which implied there might be a false negative result of QTF for silico-tuberculosis, and there was other possibility of negative QFT results when considering the individual immunity status. Study showed that the expression of PD-1 on lymphocytes reduced the sensitivity of QFT-GIT [23]. Thus, QFT might not be helpful in the diagnosis of tuberculosis for silicosis people.

Due to long-term exposure to a large amount of silica, the lung function of silicosis people appeared irreversible and severe damage, as well as many complications [24]. According to the imaging features, different degrees of silicosis were divided into primary silicosis, secondary silicosis and tertiary silicosis [25]. However, the results of our study showed no statistical difference for LTBI rates among different silicosis grades. A recent study conducted in China found the stage one silicosis was in association with LTBI as well [17].

In our study, we also found that current drinking and tunnelling work were risk factors for LTBI in silicosis participants. The result for drinking was different from the Zhejiang study in China, which showed no statistical difference between drinkers and abstainers compared with those who never drank [17]. In different work categories, we found the latent infection rate for tunneling workers was the highest compared to other works, which was similar to previous studies [26]. Meanwhile, we need to admit that some other occupations might have high LTBI, as the working environment and living conditions of the miners were different.

Our study had several limitations. Firstly, the enrollment was based on regular physical examination of silicosis participants, and they were capable of completing questionnaires and physical examination independently, suggesting these silicosis people were under a healthy physical condition. The LTBI status for those silicosis participants with poor health conditions was not evaluated in this study. Secondly, although we checked the information provided by the participants against their health records, potential information bias would not be excluded; Third, the sample size was relatively small and multiple center evaluation of LTBI among the miner workers are warranted.

## Conclusion

This cross-sectional study showed that the LTBI rate among the retired silicosis people in eastern China was higher than the corresponding age group people in China. Drinking alcohol, and tunneling work were independent risk factors for LTBI among silicosis participants. Considering the high proportion of tuberculosis for silicosis participants, LTBI screening and preventive treatment should be actively carried out in this risk group of silicosis people.

## Data Availability

Data is provided within the manuscript or supplementary information files.
